# Preparation of peroxidase and phenolics using discarded sweet potato old stems

**DOI:** 10.1038/s41598-019-40568-9

**Published:** 2019-03-06

**Authors:** Liu Yang, Yi Xi, Xiang-Yu Luo, He Ni, Hai-Hang Li

**Affiliations:** 1 Guangdong Provincial Key Lab of Biotechnology for Plant Development, School of Life Sciences, South China Normal University, Guangzhou, 510631 China; 2 School of Life Sciences, Huizhou University, Huizhou, 516007 China

## Abstract

Sweet potato (*Ipomoea batatas* L.) is the sixth most important food crop in the world. The industry discarded huge amount of sweet potato stems, rich of peroxidases and phenolics. A simple procedure was developed to make peroxidases and phenolics from sweet potato old stems. Dried stem powder was loaded into columns with water and eluted sequentially with water and 50% ethanol. Peroxidases (91%) were extracted in 5.5-fold water extracts and 87% phenolics were extracted in 4.4-fold ethanol extracts. Purified peroxidases powder was yielded at 3.1 g (8.6 unit/mg) per kilogram stems by PEG6000/Na_2_SO_4_ aqueous two-phase purification from the water extracts (93.2% recovery), followed by ethanol precipitation and vacuum freeze-drying. The purified peroxidase had high activity in transforming tea catechins into theaflavins. Phenolics powder containing 43% phenolics and 27% flavonoids was yielded at 76.9 g per kilogram stems after vacuum-concentrating the ethanol extracts. This method can make valuable functional products using the sweet potato waste.

## Introduction

Sweet potato [*Ipomoea batatas* (L.) Lam] is the sixth most important food crop in the world with annual tuberous root production of more than 100 million tons. The crop is tolerant of diseases, pests and abiotic stresses and uses little pesticides in cultivation^1^. Recent studies show that sweet potato contains many functional components such as phenolics, flavonoids and dietary fiber, which are important for human health^2^. However, the sweet potato cultivation industry produces about 70 million tons of vine stems and leaves annually which are mostly discarded in the field ridge. The young stems and leaves are rich in nutrition and are widely used as vegetable food and health foods^3,4^, while the old stems and leaves that cover most of the aerial part of sweet potato are largely ignored. It is of great significance to find out the value and use of the sweet potato old stems, in order to improve the economic value of the sweet potato crop and to reduce the environmental wastes.

Many studies indicated that the sweet potato stems and leaves contain high amount of phenolic compounds^4^. Ishida *et al*.^5^ reported that the contents of total phenolic compounds in leaves, petioles, stems and roots are 90, 45, 90, and 180 mg/100 g in the cultivar Koganesengan, and 356, 126, 197, and 154 mg/100 g in Beniazuma, respectively. Several anthocyanins and other flavonoids, chlorogenic acid and its structural analogues have been identified in the phenolic extracts of sweet potato stems and leaves^3,6^. Sweet potato stem and leaf extracts have been demonstrated to have multiple physiological and health functions^4,6–8^ and potential uses in food and medicinal industries^8,9^.

Peroxidase (EC 1.11.1.x) has been widely used in several industries^10^. It is the key enzyme in enzyme-linked immunoassay^11–13^, and has been used as biosensor to accurately detect physical, chemical and biological signals^14^. Peroxidase can be used to treat industrial waste waters and remove toxic pollutants by enzyme-catalyzed oxidation and polymerization, or by converting toxic materials into less harmful substances such as phenols, polycyclic aromatic hydrocarbons and aromatic amines^15,16^. Peroxidase can also be used in many manufacturing processes like adhesives, computer chips, car parts, and linings of drums and cans. In food industry, peroxidase is an excellent food additive to whiten the flour. Like polyphenol oxidase^1^, peroxidases can catalyze the transformation of catechins into theaflavins and improve the quality of black tea^17,18^.

In our early screening of polyphenol oxidases and peroxidase from natural biological resources, we found that the sweet potato stems had high peroxidase activity, specifically in the old stems. We developed a simple approach to simultaneously extract and purify peroxidases and phenolics from sweet potato old stems based on the newly developed column chromatographic extraction with gradient elution^19^ and the aqueous two-phase purification. The work provides a practical method to produce valuable functional products using sweet potato waste.

## Materials and Methods

### Plant materials and reagents

Tuberous roots of purple, yellow, red, and white (flesh) sweet potatoes (*Ipomoea batatas* L.) were purchased from the Jiangnan Market of Agricultural Products, Guangzhou, China. The aerial parts (vine stems and leaves) of sweet potato were obtained from a local farmer. The plant materials were cleaned with water and used directly for extraction of fresh material or for the extraction of dried material after air-dried for three more days. Except for the screening of peroxidase activity in different kinds and different parts of sweet potato which used fresh plant materials, air-dried old stems and leaves of the white (flesh) sweet potato were used in all other experiments. Young stems means the top 10 cm stems with young leaves, and the other part of the stem is called the old stem. Air-dried old stems were pulverized with a grinder and then sieved through 40 mesh sieve. The powder smaller than 40 mesh was used for extraction of peroxidases and phenolics.

Protein marker is purchased from Takara Co. (Shanghai, China). Bovine serum albumin, Coomassie brilliant blue R-250 and G250, and rutin were purchased from Sigma-Aldrich (St. Louis, MO, USA). HPLC-grade methanol was purchased from Honeywell (Maurice, NJ, USA). Other chemical and biochemical reagents are analytical or biochemical grade and were purchased from local supplier.

### Quantitative analysis of total proteins, total phenolics and total flavonoids

The protein content was determined by the method of Bradford^20,21^ with Coomassie Plus Protein Assay Reagent (Pierce Chemical Co., Rockford, IL, USA) and bovine serum albumin as the reference protein standard^22^.

Total phenolics content was determined by the Folin-Ciocalteu method using gallic acid as the reference standard. The content of total flavonoids was determined by the spectrophotometric method as described by^23^, using rutin as the reference standard^24^.

### Analysis of peroxidase activity

Peroxidase activity was determined by the pyrogallol oxidation method in the presence of H_2_O_2_. A unit of enzyme activity (U) was defined as the amount of oxidation of pyrogallol into purpurogallin at pH 6 in 20 seconds, according to Chance and Maehly’s report^25^.

In a test tube, 0.32 mL pyrogallic acid (5%, w/v), 0.16 mL H_2_O_2_ (0.5%, v/v), 0.32 mL potassium sulfate buffer (0.1 mol/L, pH 6.0) and 2.2 mL distilled water were added. The reaction solution was fully mixed and poured into a colorimetric cuvette. The absorbance at 420 nm was recorded as the blank control. Then 0.05 mL enzyme solution was added into the cuvette, mixed by pipetting up and down several times, and recorded immediately the absorbance at 420 nm for 5 min. The enzyme activity was calculated as peroxidase activity (unit/g dry material) = [(sample ΔOD − control ΔOD) × 3 × df × V]/(12 × 0.05 × W). In this equation, ΔOD represents the difference of absorbance at 420 nm per 20 seconds over the 5 min period; 3, the volume of reaction system; df, dilution factor; V, the total volume of enzyme solution extracted from the material; 12, the extinction coefficient of 1.0 mg/mL purpurogallin at 420 nm; 0.05, the volume of enzyme solution added to the reaction; W, dry weight of material used for extraction.

### Electrophoretic and bio-autographic analysis of peroxidases

Enzyme samples were separated and analyzed by native polyacrylamide gel electrophoresis (PAGE) in a Bio-Rad Mini Gel system according to the method of Laemmli^26^. Samples were mixed with glycerol and bromophenol blue and loaded onto a polyacrylamide gel (5% stacking and 10% separating gels) and electrophoresed at 80-V constant current until the bromophenol blue moved to ca. 1 cm from the gel bottom. For protein analysis, the gel was rinsed in distilled water and stained in Coomassie blue solution after electrophoresis. For bio-autographic analysis of peroxidases, the gel was stained in acidic benzidine solution with 3% H_2_O_2_ based on the report of Huang and Weng^27^.

### Maceration extraction of peroxidases

For the screening of peroxidase activity, fresh plant materials were used to extract peroxidase. 20 g fresh plant materials were cut into small pieces and homogenized. After standing for 1 h on ice water, the homogenate was centrifuged at 3000 g, and the supernatant was used to detect peroxidase activity. For the determination of extraction conditions of peroxidases from old stems, the dried stem powder was used.

### Column chromatographic extraction of peroxidases and phenolics from old stems

Simple column chromatographic extraction (CCE) is based on the method of Ni *et al*.^28^ and CCE with gradient elution to simultaneously extract all soluble substances in plant materials is based on Han *et al*.’s method^19^. For determination of optimal solvents, minimum time for the target substances fully dissolved, and the minimum volume (MV) of solvent for the material fully absorbed, 1.0 g materials was added into 10 mL solvent and macerated with shaking for up to 4 h, the enzyme activity in the solution or the solvent absorbed were monitored.

In analytical extraction experiments, 24 g stem powder was loaded with 5.0-fold water (1.0 MV) into a column (Φ2.7 cm × 35 cm) at a diameter to height ratio of 1: 10. After standing for 1 h until peroxidase was fully dissolved, the column was sequentially eluted with another 0.5-fold water, followed by 4.4-fold of 50% ethanol, and finally with water until preset volumes of eluents were collected, at the flow rate of 1.0 MV per hour. Eluent of 5.5-fold water extracts was collected as peroxidase extracts and 4.4-fold 50% ethanol extracts was collected as phenolics extracts. The aqueous peroxidase solution was used for enzyme purification directly or after ultrafiltration to its 1/5 volume through 10 kilo Dalton (kDa) molecular cut-off membrane. The aqueous ethanol solution was vacuum-evaporated to dry. Phenolics powder was obtained and the ethanol was recovered for reuse. In scaled-up extractions to prepare samples of extracts, 480 g material was used to prepare extracts of peroxidase and phenolics.

### Separation of peroxidases by adsorption and precipitation

In adsorption experiments, 1.0 g adsorbent was added into the enzyme solution at pH 4.0 or pH 10.0, adsorbed for1.0 h with gentle stirring, and centrifuged at 3000 g for 10 min. The precipitate was dissolved with PBS buffer (pH 6.0) and the protein contents and peroxidase activity of both phases were detected.

Different precipitation methods, including isoelectric point precipitation (pH 3.0–10.0), ammonium sulfate precipitation (10–80%) and ethanol precipitation (20–80%) were tested for the efficiency of precipitating and separating peroxidases, as previous reported^29^.

### Separation of peroxidase by aqueous two-phase extraction

The aqueous two-phase extraction is based on the method by Asenjo and Andrews^30^ and Glyk *et al*.^31^. Stock solutions of 50% polyethylene glycol (PEG) 6000 and 25% Na_2_SO_4_ were added into a scale centrifuge tuber at preset volumes and proportion. After fully mixed, peroxidase solution was added into the tube and water was added to the preset total volume. The tuber was vortexed for one min and centrifuged at 5000 g for 5 min at room temperature to stratify the two aqueous phases. The volumes of the two phases were read through the scale on the tube, their volume ratio was calculated, and the protein contents, enzyme activities and their distribution coefficient in the two phases were analyzed.

### Transformation of tea catechins into theaflavins by peroxidase

Green tea extracts were prepared as described before^32^. The theaflavin transformation and analysis of theaflavins are the same as described by Li *et al*.^1^. The reaction was conducted in a 25-mL solution of green tea extracts which contains 1.0 mg/mL EGCG, 2 mL peroxidase solution and 100 μL 30% H_2_O_2_. Reaction time was 10 min. Theaflavins were analyzed by HPLC^1^.

All experiments were repeated at least three times. Data presented are the means of three repeats ± standard error.

## Results and Discussion

### Peroxidase activities in different cultivars and parts of sweet potato

**Figure 1:**
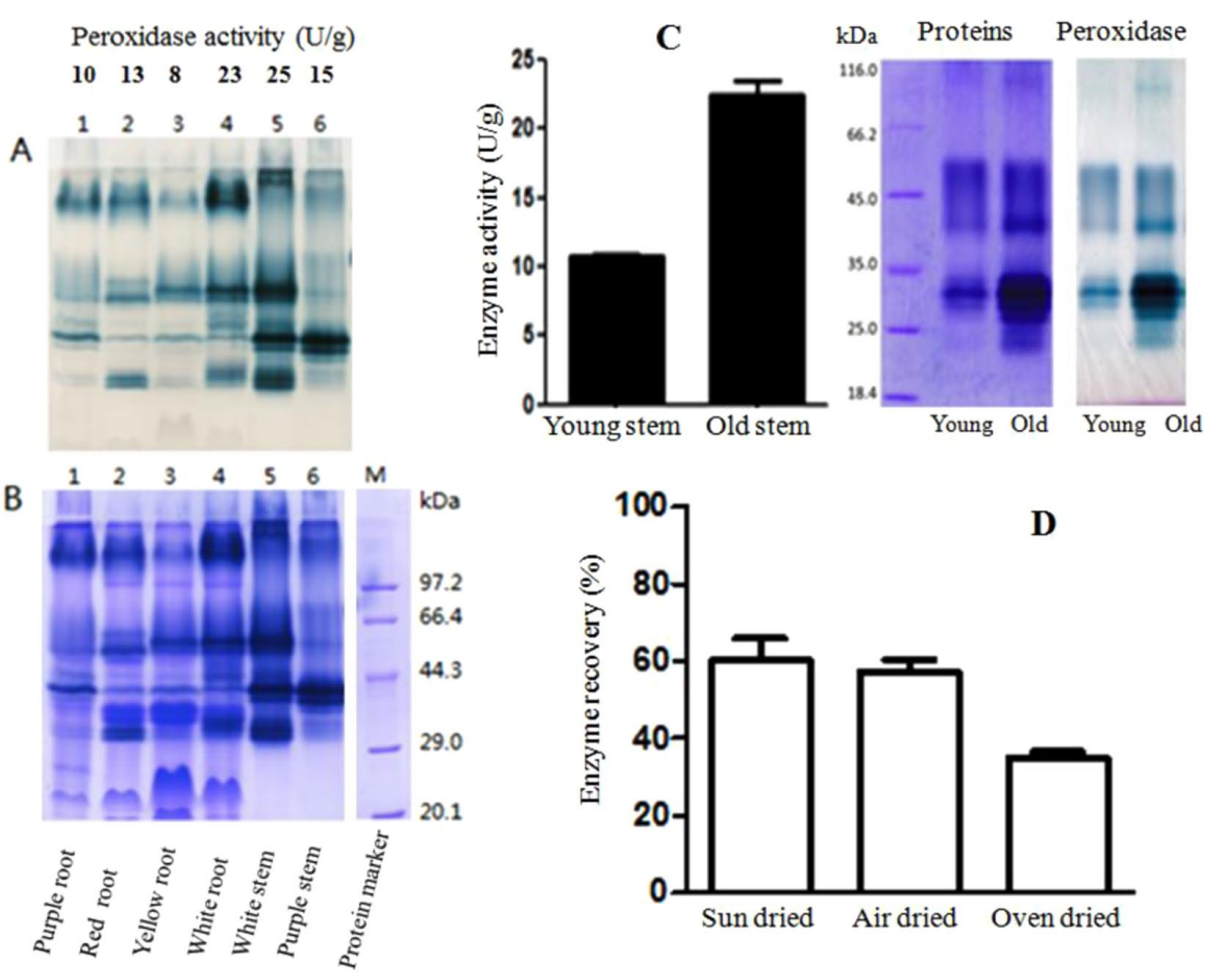
Peroxidase activity in different materials of sweet potato. (**A**,**B**), Peroxidase (**A**) and proteins (**B**) in different kinds of sweet potato tuberous roods and stems; (**C**) peroxidase and proteins in young and old stems of white sweet potato; (**D**) retained peroxidase activities in the old sweet potato stems after dried in different conditions.

In the pre-screening of peroxidase in different plant species, it was found the sweet potato had high peroxidase activity. The enzyme activity in different cultivars and organs of sweet potato were analyzed. The results are shown in Fig. 1. In the tuberous roots of purple, red, yellow and white flesh sweet potato, the white sweet potato showed the highest peroxidase activity (23 U/g), followed by the red (13 U/g), purple (10 U/g) and yellow (8 U/g) cultivars. Similar peroxidase activity distributions were also found in the corresponding stems, the highest enzyme activity was found in the aerial part of white sweet potato (25 U/g), which is higher than in the tuberous roots of the white sweet potato (Fig. 1A,B).

The stem of white sweet potato was divided into two parts, the top 10 cm young stem with young leaves which is rich of nutrient and is an excellent vegetable food^3^, and the old stem, the other part of stem with old leaves. The peroxidase activity was analyzed separately. As shown in Fig. 1C, the old stems (22.5 U/g) had much higher peroxidase activity than the young stems (10.7 U/g). The so far useless old stem was used to extract peroxidase and phenolics.

Many plant crops are harvested in the short period of time in a year, it is difficult to processing all the materials within the harvesting time. The effect of drying the sweet potato old stems on peroxidase activity was evaluated. The stem material was dried in three different ways, dried in the sun (sun-dried) for 3 days, dried in a shaded and ventilated place (air-dried) for 5 days, and dried in an electric oven (oven-dried) at 55 °C for 24 h. The peroxidase activity in the dried materials were extracted and assayed. As shown in Fig. 1D, peroxidase activity retained 60%, 58% and 35% over the fresh material after dried in the sun, in the air and in the oven, respectively. Although a significant loss of the peroxidase activity (ca. 40% or more) in the sun-dried and air-dried materials, millions of tons of sweet potato stem is still a huge resource for preparation of peroxidases. The air-dried sweet potato stems, a common dry stems for storage by traditional farmers, were used to prepare peroxidases and phenolics.

### Extraction of peroxidases by the traditional maceration method

In the following experiments, air-dried sweet potato old stems were used to extract peroxidases and phenolic compounds. However, fresh stems can also be used directly for the extraction by cutting and homogenizing the materials using the adjusted volumes of solvents based on the material dry weight (data not shown). Two extraction methods, the traditional maceration method and the newly developed column chromatographic extraction (CCE), were tested and compared to extract peroxidase and/or together with phenolics from dried stem material of sweet potato.

**Figure 2:**
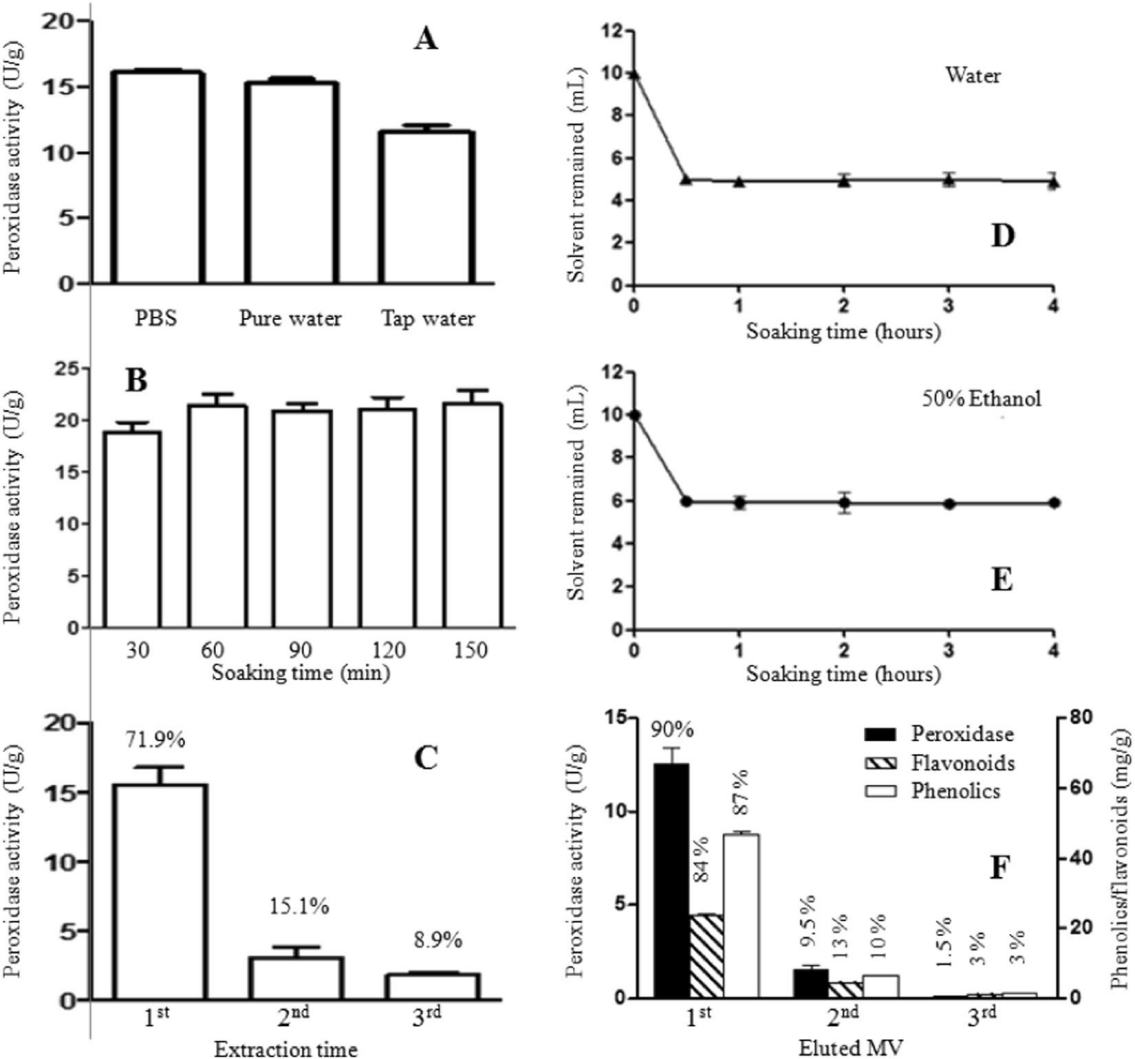
Extraction of peroxidase and phenolics from old stems of white sweet potato. (**A**) Extraction peroxidase with different aqueous solution; (**B**) dissolution of peroxidase in pure water; (**C**) extraction times of peroxidase by the maceration method; (**D**) water absorption by the sweet potato stem material; (**E**) absorption of 50% ethanol aqueous by the sweet potato stem material; (**F**) extraction of peroxidase, phenolics and flavonoids by the column chromatographic extraction with gradient elution.

Three solvents, phosphate buffer saline (PBS), pure water, and tap water were tested for the extraction efficiency of peroxidases. As shown in Fig. 2A, the extraction efficiency with PBS (16.5 U/g) is slightly higher than that with pure water (15.6 U/g). But the extraction was significant lower in the extraction with tap water (11.8 U/g). Pure water was selected for the extraction of peroxidases. The time for peroxidases of stems fully dissolved in the pure water was tested. The result indicated that the dissolution of peroxidase from the stem to the pure water reached the highest after soaked for 60 min (Fig. 2B).

The stem material was repeatedly extracted for three times by the maceration method with 10-fold pure water for 60 min each extraction. The peroxidase activities in the 1^st^, 2^nd^ and 3^rd^ extractions were 71.9%, 15.1% and 8.9%, respectively, over the total enzyme activity in the material. As plant materials are usually extracted once considering the costs and other factors in the industry, the maceration method had 71.9% extraction efficiency.

### Simultaneous extraction of peroxidases and phenolics by the column chromatographic method

Column chromatographic extraction (CCE) is a recently developed method for efficient extracting functional substances from plant materials^28^. By a gradient elution with different solvents, all soluble substances in plant materials can be completely extracted^19^. This method was tested to simultaneously extract peroxidase and phenolics in the sweet potato stems.

In addition to the extraction solvent and dissolution time of peroxidase determined in the above section, the minimum volumes (MV) of solvents for the material fully absorbed were determined. Water is efficient to extract peroxidase and 50% ethanol can efficiently extract phenolics and or flavonoids. The minimum volumes (MVs) of water and 50% ethanol required for material fully absorbed are determined to be 5.0-fold and 4.0-fold over the material dry weight (v/w), respectively (Fig. 2D,E).

First, peroxidases and phenolics were extracted from the sweet potato stem material independently by CCE method. For peroxidase extraction, the material was loaded with 5.0-fold (1 MV) water into the column and eluted with water. Three fractions, each with 5.0-fold or 1.0 MV of eluent were collected. Peroxidase activities in the three fractions were 90.0%, 9.5% and 1.5% over total enzyme activity in the material, respectively (Fig. 2F). Similar procedure was tested to extract phenolics, the material was loaded into a column with 4.0-fold of 50% ethanol and the column was eluted with 50% ethanol. Three factions of eluent each with 4.0-fold volume (v/w) were collected, phenolics in the three fractions were 87%, 10% and 3%, and flavonoids in the three fractions were 85%, 12 and 3%, respectively, over their total amounts in the stem material (Fig. 2F). The results indicated that, in the CCE extraction, the first MV of water eluent (5.5-fold) contains more than 90% peroxidase, and the first MV of 50% ethanol eluent (4.4-fold) contains more than 85% phenolics and flavonoids. The extraction efficiencies of CCE is significantly higher than that of the traditional maceration method (71.9% using 10-fold solvent).

The CCE method with gradient or segmental elution was used to simultaneously extract peroxidase, phenolics and flavonoids. The stem material was loaded with 5.0-fold water in a column and sequentially eluted with 0.5-fold water and 4.5-fold 50% ethanol, followed by water to press out the ethanol solution. Eluents of 5.5-fold water extracts (containing peroxidase) and 4.4-fold of 50% ethanol extracts (containing phenolics and flavonoids) were separately collected. Peroxidases, phenolics and flavonoids were extracted by 91.2%, 87% and 89%, respectively. The extraction efficiencies of the three groups of substances by the CCE method with gradient elution were much higher than that of the traditional maceration method. The CCE method with gradient elution was also tested in scaled-up experiments to prepare a larger volume of water extracts and ethanol extracts (data not shown).

The organic extracts of phenolics were vacuum-evaporated to dry. Phenolics powder of 76.9 ± 5.0 g per kilogram of stem material was obtained and the ethanol was recovered for reuse. The phenolic extracts contained 43 ± 4% total phenolics and 27 ± 3% total flavonoids. The water extracts of peroxidases were used to prepare purified peroxidases. In large scale processing, the water extracts could be concentrated to reduce volume to its 1/3 or 1/5 volume by ultrafiltration using a 10 kDa molecular cut-off membrane without enzyme loss, before used for purification of the enzyme (data not shown).

### Purification of peroxidases by adsorption and precipitation

**Figure 3:**
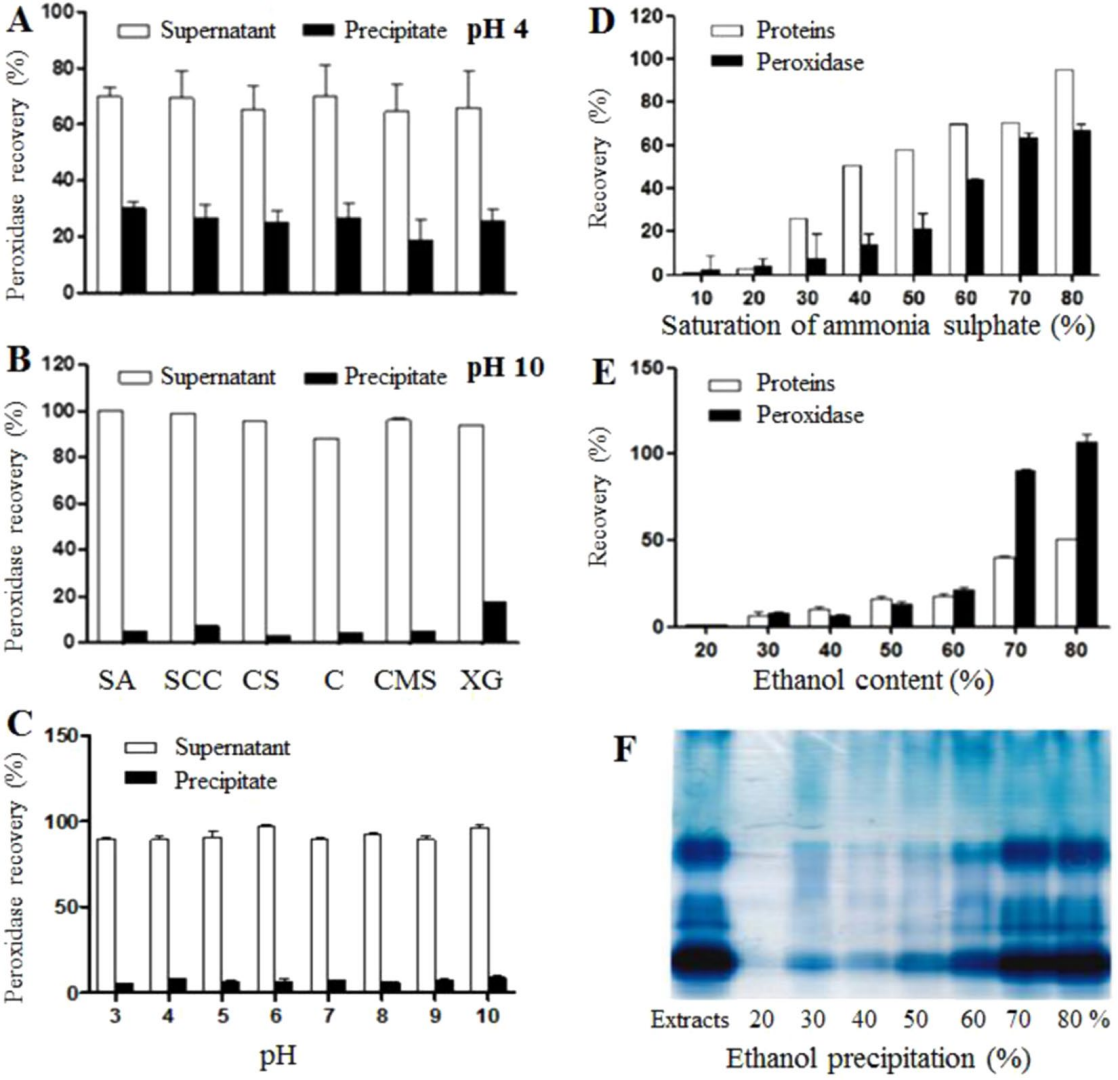
Separation of peroxidase by adsorbents (**A**,**B**) and precipitation (**C**–**F**). (**A**,**B**) Adsorption of peroxidase by six adsorbents at pH 4 (**A**) and pH 10 (**B**), SA, sodium alginate; SCC, sodium carboxymethyl cellulose; CS, cationic starch; (**C**) chitosan; CMS, carboxymethyl starch; and XG, xanthan gum. (**C**) Isoelectric precipitation of peroxidase. (**D**), precipitation of peroxidase by ammonia sulphate. (**E**) Ethanol precipitation of peroxidase; (**F**), PAGE analysis of proteins precipitated by different concentrations of ethanol.

Several common adsorption and precipitation methods were tested in order to simply concentrate and purify peroxidases in the water extracts. Six adsorbents, sodium alginate (SA), sodium carboxymethyl cellulose (SCC), cationic starch (CS), chitosan, carboxymethyl starch (CMS) and xanthan gum (XG) at 1.0% (w/v) were added into the water extracts of peroxidase at pH 4.0 and pH 10.0 and adsorbed for 1 h. The peroxidase activity in the supernatant and precipitate were analyzed. The results showed that peroxidase can not be effectively absorbed by all the six adsorbents at both pH conditions (Fig. 3A,B). Peroxidase was adsorbed by 10% to 30% by the adsorbents at pH 4.0, but couldn’t reach the expected level. And it couldn’t be adsorbed at pH 10.0. The results also indicated that the peroxidase is stable at both pH 4.0 and 10.0 conditions, without significant loss of enzyme activity.

Peroxidase couldn’t be effectively precipitated at all pH conditions tested from pH 3.0 to pH 10.0 (Fig. 3C). Peroxidase and proteins in the extracts could be effectively precipitated by ammonium sulfate at 60% saturation or higher. However, it didn’t show a differential precipitation in proteins and peroxidase and can’t separate or purify peroxidase from other proteins. In addition, the peroxidase recovery was low, and its highest recovery is only 58% at 80% saturation of ammonium sulfate (Fig. 3D). Ethanol precipitation showed better results the adsorption and other precipitation methods tested. Peroxidase recovery reached 100% in 80% ethanol precipitation, meanwhile, it can partially purify the enzyme as total proteins were precipitated only by 50% (Fig. 3E). Similar results were also observed on the PAGE analysis of the proteins precipitated by different concentrations of ethanol (Fig. 3F).

### Aqueous two-phase purification of peroxidase

**Figure 4:**
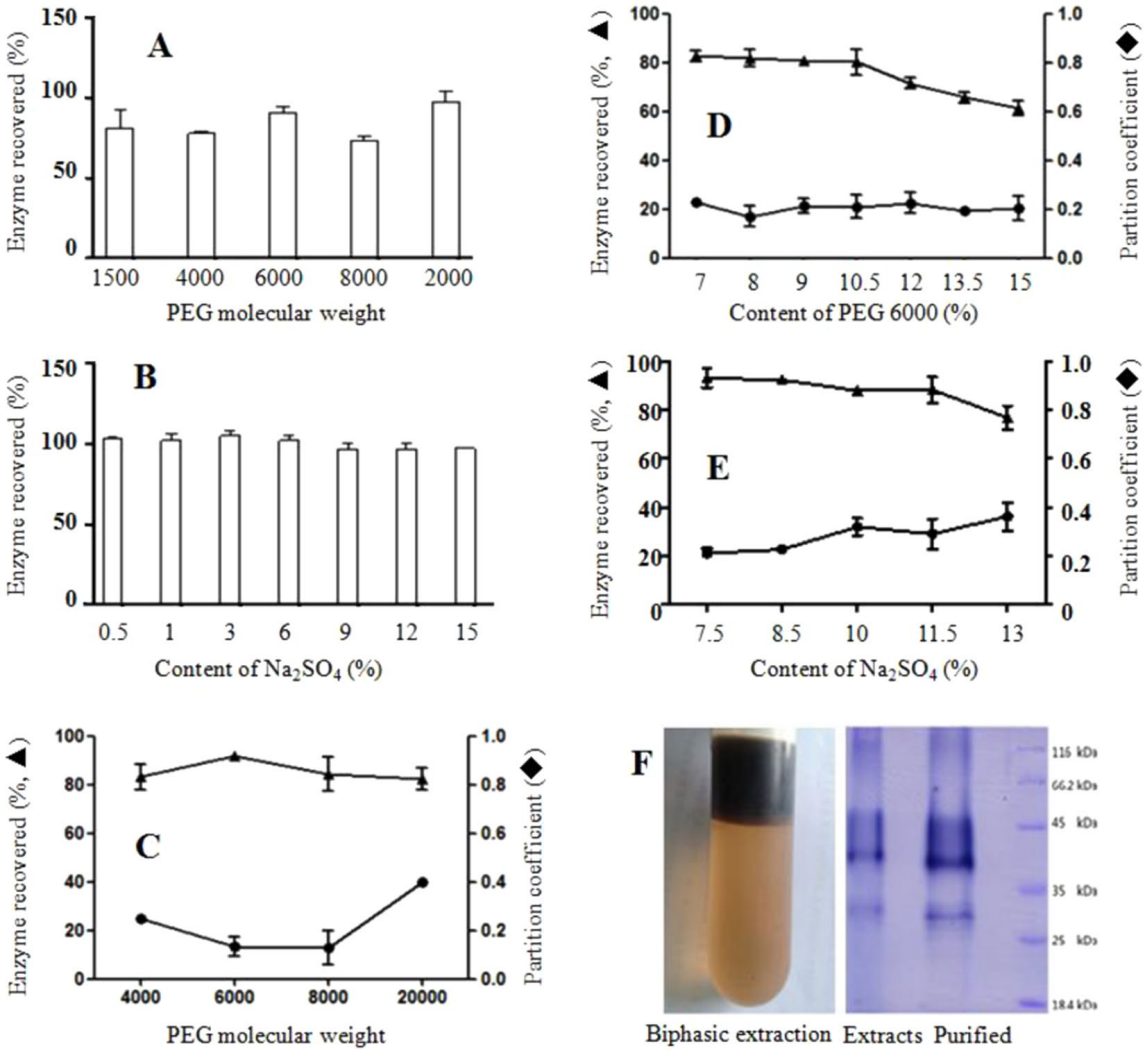
Purification of peroxidase by the PEG6000/Na_2_SO_4_ aqueous two-phase system. (**A**) Effect of different molecular weight PEG on peroxidase activity; (**B**) effect of different concentrations of Na_2_SO_4_ on peroxidase; (**C**) purification of peroxidase of Na_2_SO_4_ with different molecular weight of PEG; (**D**) effect of PEG6000 concentration on the purification of peroxidase; (**E**) effect of Na_2_SO_4_ concentration on the purification of peroxidase; (**F**), separation of peroxidase by the aqueous two-phase system and PAGE analysis of purified peroxidase.

The aqueous two-phase system composed of PEG and salts are often used to separate proteins^30,31^. The aqueous biphasic system of PEG and Na_2_SO_4_ was investigated to purify peroxidase in the extracts of sweet potato stems. The effects of different molecular weight PEG and different contents of Na_2_SO_4_ on the peroxidase activity were studied. The results indicated that PEG 1500, PEG 4000, PEG 6000, PEG 8000 and PEG 20000 at concentration of 8% caused up to 20% decrease of peroxidase activity. Among them, PEG 6000 and PEG 20000 had smaller effect on enzyme activity, which decreased the enzyme activity by 5% of less (Fig. 4A). Na_2_SO_4_ at the concentrations up to 15% did not have significant effect on peroxidase activity (Fig. 4B).

The effects of Na_2_SO_4_ (8%) with different molecular weight PEGs (8%) on the enzyme distribution between the two aqueous phases and the recovery of enzyme activity were compared. The results showed that PEG6000 and PEG8000 had better purification of peroxidases than the others, both of them had a low distribution coefficient of 0.1. The PEG6000/Na_2_SO_4_ system also had the highest of enzyme recovery of 92% and was selected as the aqueous two-phase system to purify peroxidase in the sweet potato stem extracts (Fig. 4C).

The concentrations of both PEG6000 and Na_2_SO_4_ in the PEG6000/Na_2_SO_4_ system were optimized for purifying peroxidase. When Na_2_SO_4_ content remained constant at 8%, peroxidase recovery had slight decrease when the PEG 6000 content increased from 7% to 10.5, but all with higher than 80% recovery. When PEG 6000 content was higher than 10.5%, the enzyme recovery decreased significantly. PEG 6000 at 8% also showed the lowest peroxidase distribution coefficient or best purification of peroxidase (Fig. 4D). When PEG6000 remained constant at 8%, the recovery of peroxidase had slow decrease as Na_2_SO_4_ content increased from 7.5% to 10.5%, but significant decrease as Na_2_SO_4_ content continuously increased to higher than 10.5% (Fig. 4E). At the concentrations of 8% and 7.5% Na_2_SO_4_, peroxidase recovery was the highest (93.2%), and the distribution coefficient of peroxidase in the two phases was the lowest (0.21) (Fig. 4E). The results indicated that PEG6000/Na_2_SO_4_ system at the concentration ratio of 8%: 7.5% is the best aqueous two-phase system for purifying peroxidase of sweet potato stems. The protein analysis of purified peroxidase by the PEG6000/Na_2_SO_4_ aqueous biphasic system and the bio-autography of PAGE are shown in Fig. 4F.

To decrease the volume of the aqueous two-phase system and increase the enzyme concentration in the solution in scaled-up preparation, dry powders of 8% PEG6000 and 7.5% Na_2_SO_4_ were added directly into the enzyme solution, after fully dissolved and fractionated, two aqueous phases with clear separation were obtained. The upper dark brown PEG6000 phase contained 15% enzyme activity, and the lower light yellow Na_2_SO_4_ solution contained 82% peroxidase. The peroxidase in the Na_2_SO_4_ solution was purified 6.32 folds. After precipitation in 80% ethanol and vacuum freeze-dried of the enzyme in Na_2_SO_4_ solution, peroxidase powder was obtained at the yield of 3.5 g from one kilogram of sweet potato old stems with 8.6 U/mg peroxidase activity.

### Transformation of catechins to theaflavins by the peroxidase

**Figure 5:**
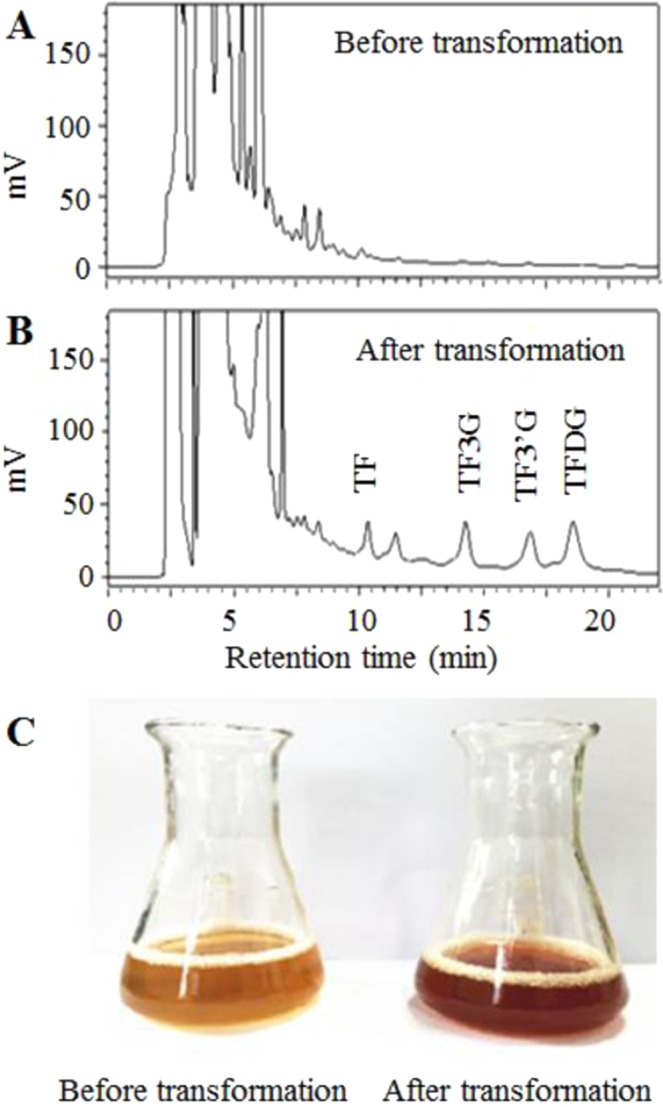
Transformation of catechins into theaflavins by the peroxidase prepared from sweet potato old stems. (**A**) HPLC analysis of green tea extracts (without theaflavins); (**B**), HPLC analysis of peroxidase transformed green tea extracts (containing theaflavins); (**C**), photogram of green tea extracts before and after transformation by peroxidase.

**Figure 6:**
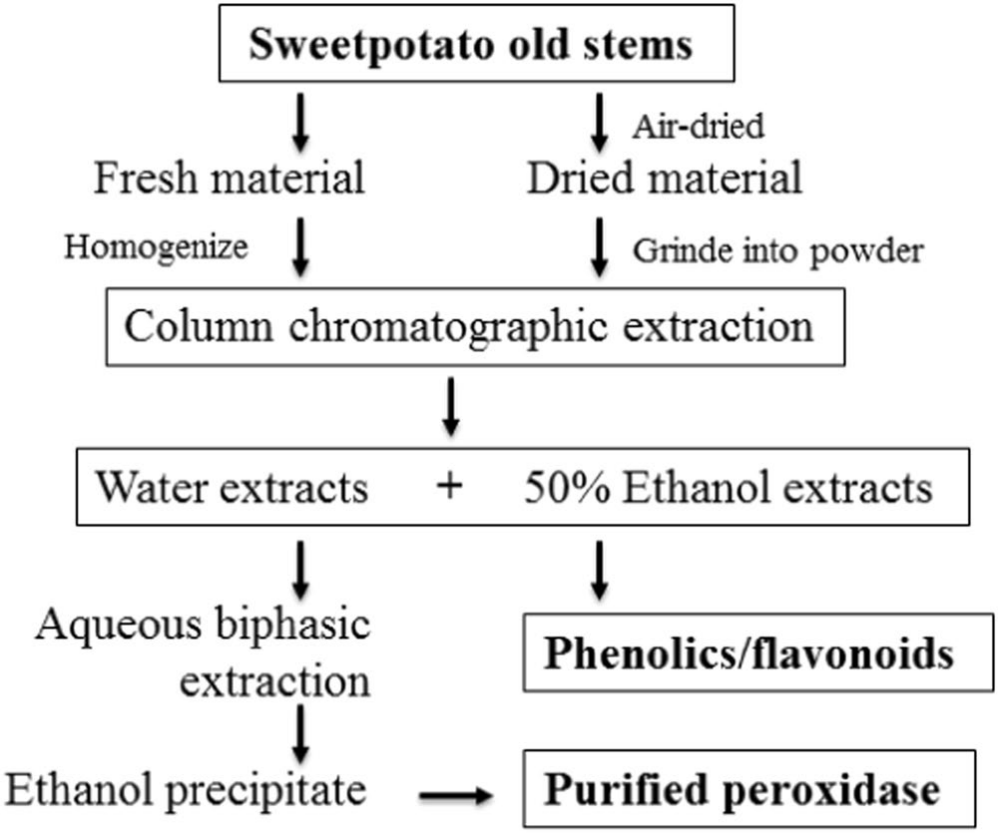
Flowchart of preparation of peroxidase, phenolics and flavonoids from the old stems of white flesh sweet potato.

Enzymatic transformation of catechins in green tea extracts into theaflavins was investigated using the purified peroxidase from sweet potato old stems (Fig. 5). HPLC analysis of the transformed tea solution indicated that peroxidase had the same function and similar level of activity as the polyphenol oxidases in transformation of theaflavins^1^. The results indicated that the peroxidase prepared has great potential in preparing functional products of theaflavins and in industrial processing of black tea. The whole processes of extracting peroxidase, phenolics and flavonoids and purifying peroxidase are outlined in Fig. 6.

## Conclusion

Sweet potato is the sixth most important food crop in the world (Islam, 2006). However, the industry produces huge amount of sweet potato stems and leaves discarded as waste. In this work, a simple procedure (Fig. 6) was developed to simultaneously extract peroxidases, phenolics and flavonoids from sweet potato old stems by the CCE method with gradient elution. Peroxidase and phenolics in the dried or fresh sweet potato stems were effectively extracted with water and 50% ethanol at the extraction efficiencies of 91% and 98.7%, respectively. The peroxidase in the water extracts were purified by the PEG6000/Na_2_SO_4_ aqueous two-phase system at the recovery of 93.2%. After ethanol precipitation and vacuum freeze-drying, purified peroxidase powder at the yield of 3.05 g/kg stems (8.6 U/mg) were obtained. The purified peroxidases had high activity to transform catechins into theaflavins. The 50% ethanol extracts were vacuum-evaporated to dried powder and 76.9 g powder which contains 43% total phenolics and 27% total flavonoids was obtained from 1.0 kg sweet potato stems. The residue after extraction can be used to prepare dietary fiber, fish and animal feeds, organic fertilizer etc. This work provides a practical approach to produce valuable functional products using the bulk waste of sweet potato, which has great potential to increase the economic profit and resource utilization of the crop and decrease the environmental waste.
